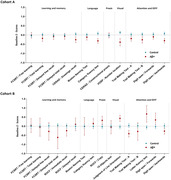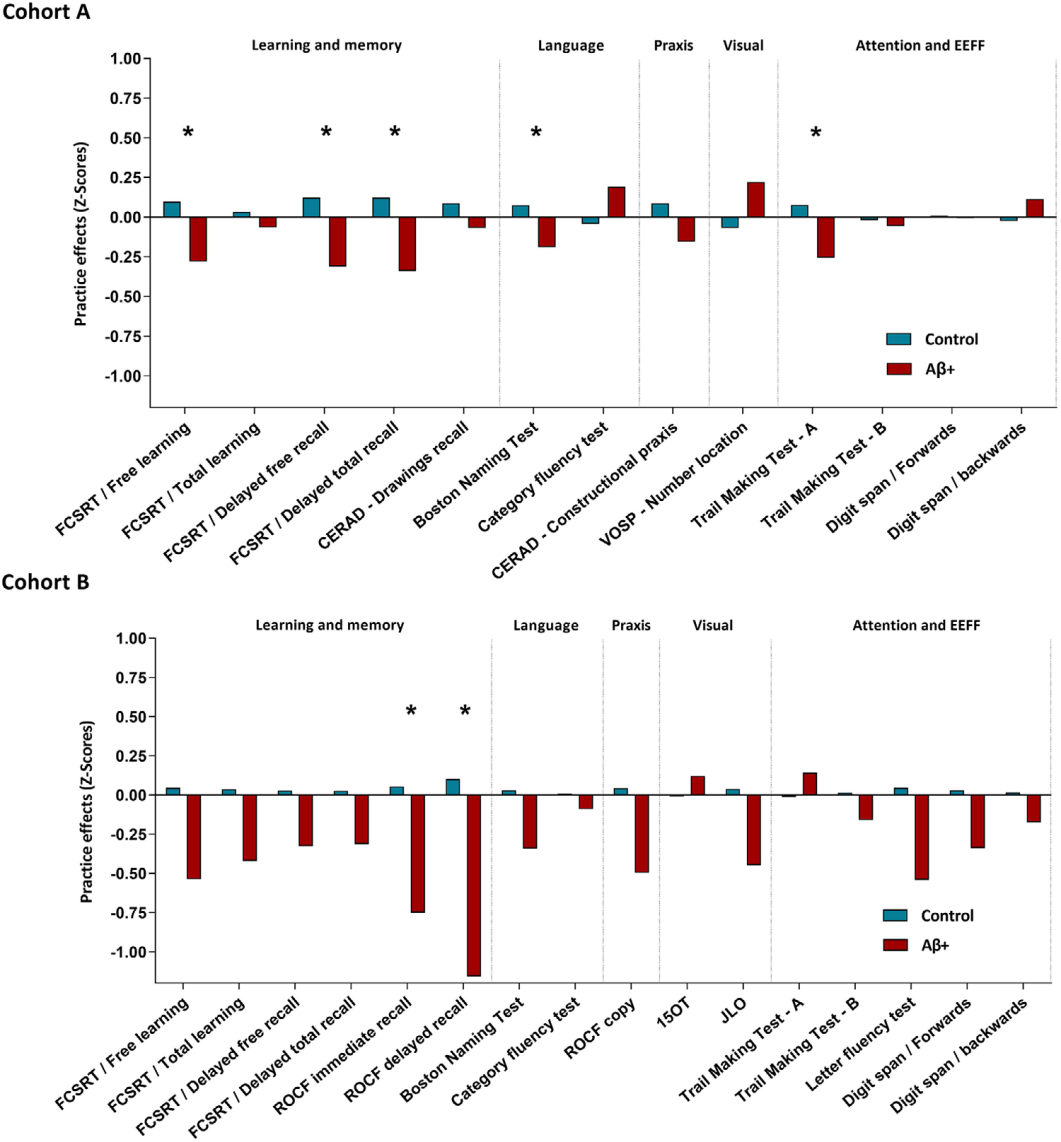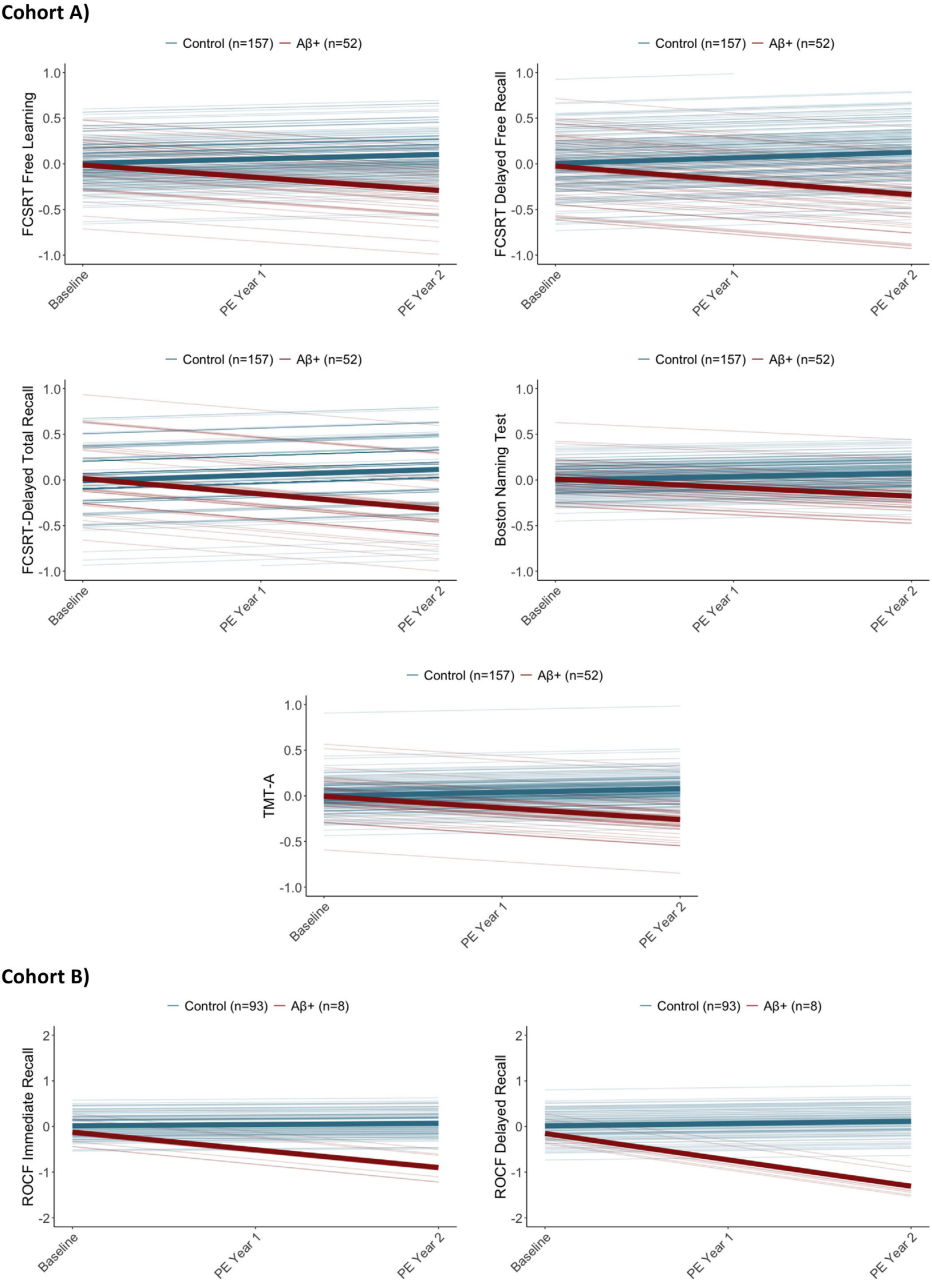# Decreased practice effects in preclinical Alzheimer’s disease: a multicenter, longitudinal, cohort study

**DOI:** 10.1002/alz.087332

**Published:** 2025-01-03

**Authors:** Adrià Tort‐Merino, Agnès Pérez‐Millan, Neus Falgàs Martínez, Sergi Borrego‐Écija, Núria Guillén, Jordi Sarto, Diana Esteller, Bea Bosch, Magdalena Castellví, Jordi Juncà‐Parella, Andrea Val‐Guardiola, Guadalupe Fernandez‐Villullas, Anna Antonell, M Belen Sánchez‐Saudinós, Sara Rubio‐Guerra, Nuole Zhu, María García Martínez, Ana Pozueta, Ainara Estanga, Mirian Ecay, Carolina Lopez, Mikel Tainta, Miren Altuna, Eloy Rodríguez Rodríguez, Pascual Sanchez‐Juan, Pablo Martinez‐Lage, Alberto Lleo, Juan Fortea, Ignacio Illán‐Gala, Mircea Balasa, Albert Lladó, Lorena Rami, Raquel Sánchez‐Valle

**Affiliations:** ^1^ Hospital Clínic de Barcelona ‐ Fundació de Recerca Clínic Barcelona – IDIBAPS ‐ University of Barcelona, Barcelona, Catalonia Spain; ^2^ Alzheimer’s disease and other cognitive disorders Unit. Hospital Clínic de Barcelona. Fundació de Recerca Clínic Barcelona – IDIBAPS. University of Barcelona, Barcelona Spain; ^3^ Alzheimer’s disease and other cognitive disorders Unit. Hospital Clínic de Barcelona; FRCB‐IDIBAPS; University of Barcelona, Barcelona Spain; ^4^ Memory Unit, Department of Neurology, Hospital de la Santa Creu i Sant Pau, Barcelona Spain; ^5^ Hospital de la Santa Creu i Sant Pau, Barcelona, Barcelona Spain; ^6^ Neurology Department, Hospital Universitario Marqués de Valdecilla – IDIVAL – University of Cantabria ‐ CIBERNED, Santander, Cantabria Spain; ^7^ Hospital Universitario Marqués de Valdecilla – IDIVAL – University of Cantabria, Santander, Cantabria Spain; ^8^ Fundación CITA‐Alzhéimer Fundazioa, Centro de Investigación y Terapias Avanzadas ‐ Osakidetza, Organización Sanitaria Integrada Debabarrena (OSI) ‐ University of Deusto, San Sebastián, Guipúzcoa Spain; ^9^ Fundación CITA‐Alzhéimer Fundazioa, Centro de Investigación y Terapias Avanzadas; Osakidetza, Organización Sanitaria Integrada Debabarrena (OSI); Department of Medicine, Faculty of Health Sciences, University of Deusto, San Sebastian, Guipúzcoa Spain; ^10^ Fundación CITA‐Alzhéimer Fundazioa, Centro de Investigación y Terapias Avanzadas;, San Sebastian, Guipúzcoa Spain; ^11^ Sant Pau Memory Unit, Department of Neurology, Hospital de la Santa Creu i Sant Pau, Biomedical Research Institute Sant Pau ‐ Universitat Autònoma de Barcelona, Barcelona Spain

## Abstract

**Background:**

Practice effects are a well‐known cognitive phenomenon that is reduced in patients with Alzheimer’s disease (AD). We aimed to investigate whether cognitively unimpaired (CU) individuals within the Alzheimer’s continuum (i.e., positive amyloid‐β biomarker) display decreased practice effects on serial neuropsychological testing.

**Methods:**

We included 310 CU from four Spanish research centers, classified into controls (n = 250) or Aβ+ (n = 60). In the main cohort (Cohort A; n = 209), participants underwent neuropsychological assessment at baseline and annually during a 2‐year follow‐up (FU_1_ and FU_2_). A “long‐term cohort” (Cohort B; n = 101) was employed to assess practice effects over longer time periods (i.e., two follow‐up sessions at years 3 and 6 from baseline). Practice effects were defined as simple discrepancy scores (SDS) by subtracting Z‐scores at FU_2_ from Z‐scores at baseline for each neuropsychological variable, and linear mixed effects models (LME) were run to assess the temporal evolution of practice effects according to the two groups.

**Results:**

There were no cross‐sectional differences between the control and Aβ+ groups in none of the neuropsychological scores at baseline (Fig. 1). The Aβ+ group displayed lower practice effects than the controls in terms of SDS in several neuropsychological outcomes (Fig. 2). In Cohort A, LME showed negative slopes by the Aβ+ group in verbal memory measures such as the free learning score (β = ‐0.37, SD = 0.12, p = 0.0034), delayed free recall (β = ‐0.43, SD = 0.15, p = 0.0047) and delayed total recall (β = ‐0.46, SD = 0.17, p = 0.0069) from the Free and Cued Selective Reminding Test; as well as in language tasks (Boston Naming Test; β = ‐0.26, SD = 0.087, p = 0.0025) and executive function measures (Trail Making Test; β = ‐0.33, SD = 0.12, p = 0.0094) (Fig. 3A). In Cohort B, similar findings were observed in visual memory measures, such as the Rey‐Osterrieth Complex Figure immediate (β = ‐0.80, SD = 0.35, p = 0.024) and delayed (β = ‐1.25, SD = 0.34, p = 0.00038) recall (Fig. 3B).

**Conclusions:**

Individuals with normal cognition who are in the Alzheimer’s continuum show decreased practice effects over serial neuropsychological testing. Our findings suggest the reduction of practice effects, particularly in memory measures, as an indicator of subtle cognitive decline in the earliest phase of the Alzheimer’s continuum and could be particularly relevant for the design and interpretation of primary prevention trials on disease‐modifying therapies.